# Acceptability and Feasibility of ActivPAL Activity Tracker for Pre-Frail African American Older Adults

**DOI:** 10.1093/geroni/igaa057.622

**Published:** 2020-12-16

**Authors:** Yi-Ling Hu, Camila Rodriguez Valenzuela, Ryan Jones, Natalie Gamalski, Heather Fritz

**Affiliations:** 1 Wayne State University, Detroit, Michigan, United States; 2 Wayne State University, Wayne State University, Michigan, United States

## Abstract

Frailty is more prevalent among African American (AA) older adults compared to their White counterparts. Physical activity (PA) interventions are effective to attenuate frailty progression. The Activpal is adhered to the thigh and allows unobtrusive PA measurement for up to 14 days. However, few studies have tested the acceptability and feasibility of activity trackers among older pre-frail AA. In this study, pre-frail AA older adults were asked to wear the ActivPAL tracker on their thigh for 7 consecutive days. Acceptability was measured by compliance. Feasibility was evaluated by the valid number of days and programing errors. Intraclass correlation coefficients were used to assess the agreement of light to vigorous PA level between ActivPAL and the Community Healthy Activities Model Program for Seniors (CHAMPS). In a sample of 28 pre-frail AA older adults (mean age= 73.21,SD= 9.37, 100% female), 24 completed the 7-day data tracking. Reasons for non-compliance included skin irritation (n=1), and fear of causing other health issues (n=3). The number of valid days was= 6.9 0.33 with 1 incident of programming error. The agreement between ActivPAL and CHAMPS was fair (ICC =0.57, [95%CI]= -.08 -.85) with most participants (61.6%) over-reporting PA on CHAMPS. The ActivPAL accelerometer was acceptable and feasible to use among pre-frail AA older adults. Communication prior to applying the accelerometer is crucial to prevent non-compliance. The results confirm prior studies, which suggest that older adults may over-report PA levels. ActivPAL offers a feasible and accurate approach to assess PA among older AA.

